# Explicating the multifunctional roles of tocotrienol and squalene in promoting skin health

**DOI:** 10.1002/ski2.448

**Published:** 2024-08-20

**Authors:** Nevvin Raaj Morgan, Kasthuri Bai Magalingam, Ammu Kutty Radhakrishnan, Mohan Arumugam, Adawiyah Jamil, Saatheeyavaane Bhuvanendran

**Affiliations:** ^1^ Food as Medicine Research Strength Jeffrey Cheah School of Medicine and Health Sciences Monash University Bandar Sunway Malaysia; ^2^ Department of Medicine Universiti Kebangsaan Malaysia Medical Centre Kuala Lumpur Malaysia

## Abstract

The skin is the largest organ in the integumentary system, protecting against various external threats, including ultraviolet exposure, heat, infections, dehydration and mechanical injuries. Skin disorders can arise from various causes, including allergic reactions or breaches in the skin barrier, which allow microorganisms or chemicals to penetrate the sweat ducts. These conditions encompass a wide range of issues, including acne, xerosis (dry skin), fungal infections, atopic dermatitis (eczema) and psoriasis. Collectively, these ailments affect a significant portion of the global population, impacting approximately one‐third of people worldwide. Additionally, oxidative stress induced by ageing and prolonged exposure to ultraviolet rays can manifest in visible alterations such as pigmentation, wrinkling and dehydration. Recent investigations have underscored the potential of natural antioxidant compounds in safeguarding skin health and combating ageing‐related changes. Tocotrienols, a subgroup of vitamin E, have garnered significant attention owing to their antioxidant and anti‐inflammatory properties. Significant amounts of tocotrienols can be found in rice bran, olive, oats and hazelnuts. Similarly, squalene, predominantly sourced from fish liver oils such as those from sharks, has been used as an emollient in cosmetic formulations. This article offers a comprehensive review of existing literature elucidating the dermatological benefits associated with tocotrienols and squalene, emphasising their roles as antioxidants, anti‐inflammatories, skin barrier protection and facilitators of wound healing. Moreover, it sheds light on contemporary research findings suggesting these compounds' therapeutic promise in managing and ameliorating various skin conditions.

## Graphical abstract

The skin is the largest organ in the integumentary system, protecting against various external threats. Skin disorders can arise from various causes, including allergic reactions or breaches in the skin barrier. Natural compounds, such as tocotrienols and squalene, have significant skin protective properties. This article offers a comprehensive review on skin protective mechanisms of tocotrienols and squalene against oxidative stress, dermatitis and inflammation.

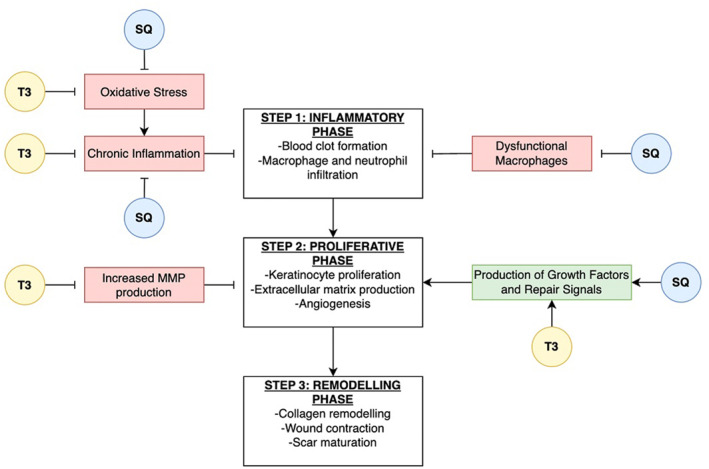

**What is already known about this topic?**

- Tocotrienols, a form of vitamin E, offer a wide range of health benefits due to their potent antioxidant properties, which aid in combating oxidative stress within the body. Conversely, squalene, a polyunsaturated lipid, serves as the primary component in skin sebum. Recognized for its antioxidant properties, squalene is commonly used in cosmetics as an emollient ingredient.

**What does this study add?**

- This research consolidates and analyzes the latest findings and advancements concerning the impact of tocotrienols and squalene on skin health. The paper emphasises experimental investigations, encompassing cell‐based and animal studies, and delves into the intricate protective mechanisms these compounds offer against various skin disorders.

## 1 INTRODUCTION

The skin is the largest organ of the human body, making up to 15% of body weight with three layers: epidermis, dermis and subcutaneous layer.^1^ The epidermal layer can be divided into five strata: basale, spinosum, granulosum, lucidum and corneum. The types of cells found in the epidermis are 95% keratinocytes and the remaining 5% of Langerhans's cells, Merkel's cells and melanocytes.^2^ The main function of the skin is to act as a barrier achieved by the outermost strata corneum, which is made up of lipids and structural proteins produced by keratinocytes, which protect the skin and prevent water loss.^3^ The skin also functions in sensations such as pain, touch, temperature, and pressure due to the sensory receptors found in the skin abundantly.^4^ Moreover, the role of skin in thermoregulation is explained through the heat trapping function of hair and sweating by sweat glands maintaining the homoeostasis of body temperature.^5^ The skin's metabolic role involves the epidermis and subcutaneous layers. Within the epidermis, vitamin D and steroid hormones are synthesised, while the subcutaneous layer functions as a reservoir for vitamin D and releases bioactive substances that modulate metabolic pathways.^6^, ^7^, ^8^

**FIGURE 1:**
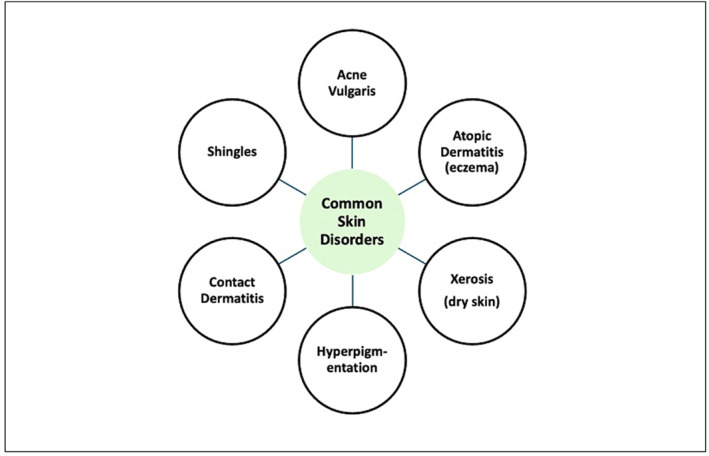
Diagram of common skin disorders.

When the components or the layers of the skin malfunction, the human skin is negatively affected, leading to common skin disorders such as atopic dermatitis (AD), acne vulgaris, xerosis and erythema (Figure 1). Most people experience skin disorders, mainly hyperpigmentation, acne and dry skin.^9^ Several therapeutic agents are available to treat skin disorders, including moisturizers, topical corticosteroids, topical calcineurin inhibitors, tyrosinase inhibitors and emollients.^10^, ^11^ Although these treatments are available for common skin disorders, research on more effective treatments is needed to better manage skin conditions, which could include the applications of naturally derived bioactive compounds.

## 2 NEW THERAPEUTIC AGENTS

In recent years, new therapeutic agents (Table 1) have emerged that may offer additional benefits for treating skin disorders. For instance, ceramides, natural lipids in the outermost skin layer, are crucial in forming a protective barrier against moisture loss. A recent qualitative review has provided evidence that applying formulations with ceramides topically can enhance skin barrier function and alleviate dry and flaky skin conditions in individuals with dermatitis.^15^, ^16^

**TABLE 1:** Newer skin therapeutics and their protective effects on skin.

New skin therapeutics	Mechanism of action	References
Urea	Enhances skin hydration by drawing moisture to the skin's outer layer and augmenting its water‐retention capacity	12
Filaggrin	Involved in the formation of protective skin barrier	13
Natural oils (e.g. argan, coconut and jojoba)	Enhances skin hydration Anti‐inflammatory	14
Botanical extracts (e.g. chamomile, aloe vera and green tea)	Antioxidant Anti‐inflammatory	14
Ceramides	Forms a protective barrier against moisture loss	15, 16
Vitamin E	Fat‐soluble vitamins with potent antioxidant activities	17
Squalene	An excellent moisturiser Keeps skin hydrated and supple	18

### 2.1 Vitamin E and squalene

Vitamin E is a fat‐soluble compound that is crucial for sustaining skin health. Its strong antioxidant potential enables it to safeguard the skin against free radicals or unstable molecules causing oxidative stress, ultimately contributing to premature ageing and various skin issues.^17^ Another phytonutrient that is gaining momentum in the field of dermatology is squalene. Squalene is a natural organic compound that is predominantly found in plant sources. Emerging studies have shown that squalene is an excellent moisturiser that helps to keep the skin hydrated and supple.^18^ This review aims to provide an overview of tocotrienols (a form of vitamin E) and squalene and highlight their key mechanisms in the amelioration of common skin ailments and promotion of general skin health.

## 3 SEARCH METHODOLOGY

The search and retrieval of the articles till December 2023 were done on electronic databases PubMed and Google Scholar. Following are the keywords used in the article search: ‘Tocotrienol’, ‘Squalene’ and ‘Skin’. The inclusion criteria of the article selection were (i) original research articles (ii) cell line, animal and human studies and (iii) articles written in English language, while the exclusion criteria were (i) books, review articles and conference papers and (ii) articles written in languages other than English.

## 4 WHAT ARE TOCOTRIENOLS?

**FIGURE 2:**
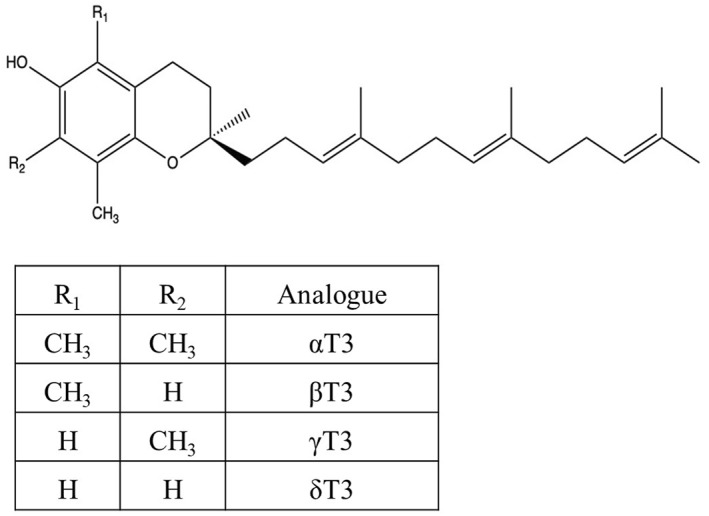
Chemical structure of tocotrienols.

Vitamin E is a collective term encompassing a group of fat‐soluble vitamins, which can be categorised into two primary types: tocotrienols (T3) and tocopherols (TP). The TP and T3 have similar chemical structure. However, the isoprenoid side chain of T3 contain three carbon double bonds, rendering it unsaturated^19^ (Figure 2). The unsaturated structure of T3 enables it to penetrate tissues and organs more effectively when compared to TP.^20^ In addition, both forms of vitamin E exist naturally in four isoforms; that is, alpha (α), beta (β), gamma (γ) and delta (δ). The distinction between α, δ, γ and β isoforms lies in the specific location and quantity of various alkyl groups (R1 and R2) on the chromanol ring (Figure 2). The primary sources of T3 are predominantly plant and seed oils, particularly palm and rice bran.^21^

The vitamin E extract from palm oil is known as the tocotrienol‐rich fraction (TRF), which contains approximately 25% alpha‐tocopherol and 75% T3.^22^ Several studies have found that T3 possesses anti‐cancer, antioxidant, anti‐inflammatory and neuroprotective properties.^21^, ^23^, ^24^ Furthermore, T3 has been shown to offer substantial protective benefits for the skin, such as safeguarding the skin by upholding skin homoeostasis through various mechanisms.

### 4.1 Tocotrienols protect against skin dermatitis

Atopic dermatitis or more commonly known as eczema, is a chronic skin inflammation that could disrupt the quality of life and has been recognized as a public health concern. This condition is marked by symptoms such as itching, dryness, scaly, flaky and redness. In addition, AD is a risk factor for the development of food allergy, asthma and allergic rhinitis in young children, and this progression is defined as ‘atopic march’.^25^ Research indicates that mutation of filaggrin encoding genes, FLG, may be the genetic predisposition, which disrupts the homoeostasis of the skin barrier as a triggering factor in AD.^26^ Adverse environmental factors, including climate change, allergens, pollutants and irritants, may exacerbate the condition.^27^ The clinical presentation of AD varies across age groups. In infants, AD often appears on the face and scalp, while flexural areas are commonly affected in children and adults. Itching is a hallmark symptom, often leading to excoriation and secondary infections.^28^ Currently, AD is managed by topical application of emollients and moisturizers to improve skin hydration. Some patients are prescribed corticosteroids and calcineurin inhibitors to alleviate skin inflammation. In addition to the treatment strategy, effective management of this condition involves identifying and avoiding potential triggering risk factors.

Skin dermatitis is primarily caused by disturbances in the tissue homoeostasis that restore the crosstalk between immune cells and microbes that colonise the skin. Inflammation is due to the activation of pro‐inflammatory mediators, such as interleukin (IL)‐1β, IL‐6, IL‐8 and tumour necrosis factor‐α (TNF‐α), which play pivotal roles in recruiting immune cells to the inflammation site to eliminate pathogens and facilitate tissue repair. For instance, IL‐1α and IL‐1β were reported to exert synergistic effects with TNF‐α and interferon‐gamma (IFN‐γ), which activates T‐cells and induces keratinocytes to produce chemokines during the inflammatory process.^29^ Furthermore, TNF‐α was reported to attract neutrophils, macrophages and skin‐related memory T‐cells and promotes tissue repair in human keratinocytes.^30^ Another crucial marker in skin inflammation are the cyclooxygenase (COX) isoforms, namely COX‐1 and COX‐2, which play important roles in the synthesis of prostaglandin E2 (PGE2), contributing to skin homoeostasis and repair.^31^ Tripp et al. validated the function, indicating that heightened expression of COX‐2 in the basal keratinocyte layer following exposure to acute ultraviolet radiation (UVR) significantly amplifying keratinocyte proliferation and apoptosis,^32^ which suggested that COX‐2 expression is crucial for keratinocyte survival and proliferation in response to oxidative stress stimuli.

Several compounds that possess skin protective properties to alleviate the pro‐inflammatory markers elicited during the inflammation process have been studied. Among these compounds include tocotrienols (T3), which was reported on the anti‐inflammatory properties of T3 and its use in dermatitis.^33^ For instance, treatment with γT3 was reported to significantly inhibit upregulation in IL‐1β, IL‐6, IL‐8, TNFα, NF‐κB and COX‐2 genes, inhibit synthesis of PGE2 and generation of reactive oxygen species (ROS) in HaCaT human keratinocyte cell line treated with squalene monohydroperoxide (SQ‐OOH).^34^ In another study, it was reported that TRF reduced phosphorylation of p38, Jun N‐terminal kinase and extracellular‐signal‐regulated kinase (ERK), which participate in the mitogen‐activated protein kinase (MAPK) signalling pathway in particulate matter (PM2.5) and induced inflammation model of HaCaT cells. Moreover, TRF also showed a downregulation of the expression of COX‐2 in human keratinocyte cells.^33^

Histamine, an aminergic neurotransmitter, is released by immune cells in response to various environmental allergens, leading to itching and inflammation. In the event of acute inflammation, immune cells, including mast cells, granulocytes, Th cells, macrophages and antigen‐presenting cells, migrate to the skin, with mast cells playing a crucial role in innate immunity. Mast cells express surface receptors with high affinity for IgE (FcεRI), allowing them to form antigen‐IgE complexes, triggering the release of histamine and activating pro‐inflammatory mediators, worsening itching symptoms on the skin. Recent research has also indicated that histamine disrupts the skin barrier by inhibiting the terminal differentiation of keratinocytes.^35^ Supplementation of T3 to picryl chloride‐induced dermatitis mice was reported to reduce scratching, skin thickening and serum histamine levels compared to the control group.^36^ The authors also found that mast cell degranulation and decreased allergic dermatitis were suppressed by T3 through the inhibition of protein kinase C activity. A recent clinical trial that evaluated the efficacy of T3 in reducing inflammation and pruritus reported that applying a T3‐rich moisturiser in children with AD showed no adverse effects with a significant reduction in pruritus intensity.^37^

### 4.2 Protective mechanism of tocotrienols against ultraviolet radiation

A common threat to human skin is UVR, and exposure to sunlight is the major source of UVR. UVR can be categorised as UVA, UVB and UVC of which the longer UVA and UVB infiltrate the ozone layer as the shortest UVC gets blocked by the ozone layer.^38^ The major negative effect of UVR on the skin is the formation of thymine dimers in the DNA.^39^ Failure to correct the thymine dimer formation will result in the mutation to persist and lead to carcinogenesis, such as basal cell carcinoma, squamous cell carcinoma and melanoma. Although the DNA repair mechanisms kick in to correct the thymine dimer formulation, this attempt can fail. A common response is the activation of the apoptotic pathway via p53 activation.^40^ The apoptotic cells release inflammatory markers, which in turn causes skin inflammation observed as redness, commonly known as sunburn. Moreover, studies have shown that DNA damage by UVR also causes higher levels of melanin production by upregulating the production of tyrosinase enzyme, which is involved in melanogenesis.^41^ The UVR on the skin can also cause hyperplasia due to the increased proliferation of keratinocytes.^42^ Considering the adverse effects of UVR, photoprotection stands out as the primary approach for UVR prevention.

There are emerging research findings that highlight significant protective properties of T3 against UVR. For instance, Weber et al. reported a substantial reduction in the skin's vitamin E levels following UVR exposure.^43^ This study found that the vitamin E levels were higher compared to the control group in individuals whose skin was pre‐treated with TRF before exposure to UVR, which suggest that applying TRF to the skin could safeguard the skin's vitamin E levels and protect against UVR‐induced oxidative stress. In another study, a noticeable reduction in sunburn severity and papilloma incidence on their skin was reported in hairless mice fed with TRF.^44^ In addition, the skin of mice fed with TRF exhibited significantly elevated T3 levels.

A previous study explored the anti‐pigmentation effects of TRF, using primary melanocytes obtained from adolescent males. In this research, melanocytes treated with TRF had significant reduction in melanin levels and reduced tyrosinase activity compared to the control group.^45^ Furthermore, the group treated with TRF also exhibited a noteworthy downregulation of the TYRP2 gene, as confirmed through real‐time RT‐PCR analysis.

In another study that used a UVR model showed that T3 effectively inhibits UVA‐induced melanogenesis by suppressing the expression of TYR, TYRP1, and TYRP2 genes.^46^ Some of the positive outcomes were observed when immortalised human keratinocytes were treated with γT3 and subsequently exposed to UVB, which include reduction in PGE2 production, decreased expression of inflammatory markers such as IL‐1β, IL‐6 and MCP‐1 and decreased COX‐2 protein levels. In addition, supplementation of HR‐1 hairless mice with γT3 attenuated UVB‐induced skin thickness, alleviated sunburn symptoms, mitigated severe hyperplasia, and reduced COX‐2 protein expression.^47^

**FIGURE 3:**
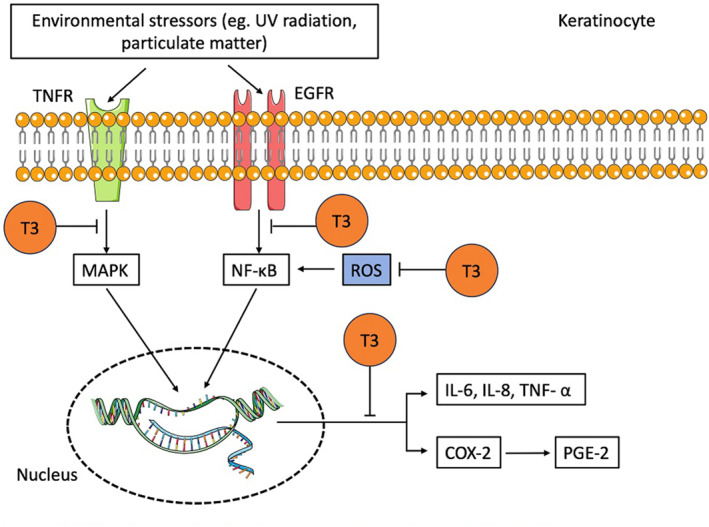
Simplified schematic diagram showing potential T3 mechanisms as an anti‐inflammatory and antioxidant agent in keratinocytes. T3 reduces ROS formation, inhibits MAPK and NF‐κB pathways, and reduces the formation of pro‐inflammatory molecules. COX‐2, cyclooxygenase 2; EGFR, epidermal growth factor receptor; IL‐6, interleukin‐6; IL‐8, interleukin‐8; MAPK, mitogen‐activated protein kinase; NF‐κB, nuclear‐factor‐kappa; PGE‐2, prostaglandin E2; ROS, reactive oxygen species; T3, tocotrienol; TNF‐α, tumour necrosis factor‐alpha; TNFR, tumour necrosis factor receptor; →, stimulation; ⊥, inhibition.

Production of ROS following exposure to UVR is one of the pathways through which UV light can harm human health. Oxidative stress can occur when ROS production surpasses the body's capacity to counteract them through antioxidant defences, that is, causing an imbalance in the cellular redox homoeostasis. The antioxidant properties of TRF have been successful in combating the effects of UVR on the skin. For instance, HaCaT cells pre‐treated with TRF prior to UVB exposure had decreased intracellular ROS levels and oxidative stress markers, including 8‐hydroxyl‐2′‐deoxyguanosine (8‐OHdG), malondialdehyde (MDA) and protein carbonyls as compared to the controls.^48^ In addition, there were reduced levels of inflammatory markers such as IL‐6, IL‐8 and COX‐2 in the TRF‐treated cells (Figure 3). In the same investigation, skin biopsies obtained from pig ear lobes were employed to assess the skin's antioxidative capacity and its radical scavenging effects. The study revealed a marked decrease in skin redness and depigmentation in the subjects treated with the TRF formulation, underscoring its substantial efficacy in mitigating the effects of UVR on the skin.^48^

### 4.3 Wound‐healing potential of tocotrienols

Wound healing involves the intricate process of regenerating and repairing the injured tissues caused by injuries. The process of wound healing consists of four phases: coagulation, inflammatory, proliferative and remodelling. During the coagulation phase, constriction of blood vessels occurs, followed by the formation of blood clots to prevent the entry of pathogens and the loss of blood.^49^ In the early stage of the inflammatory phase, infiltration of neutrophils occurs to prevent infection, while during the later stage, macrophages continue the phagocytosis process and scavenging of debris.^50^ Moreover, macrophages produce PG, PDGF, FGF, TGF‐β1 and VEGF, which stimulates angiogenesis and matrix formation.^51^ In the third stage, re‐epithelialisation occurs following keratinocyte proliferation and infiltration of fibroblasts and their proliferation.^50^ Lastly, during the remodelling stage, the wound's contraction, the epidermis maturation, the collagen content increase, and the formation of scar occurs, resulting in higher tensile strength.^52^

Deep‐partial thickness burn injuries are identified by a parched, pale or yellowish wound that affects the deeper dermis layer of the skin and leads to scar formation.^53^ An in vivo investigation conducted on rats with deep‐partial thickness burn wounds that received epidermal growth factor (EGF) and a TRF showed accelerated wound contraction and re‐epithelization in the EGF + TRF groups compared to the untreated rats.^54^ In addition, there was a decrease in neutrophils, lymphocytes and myofibroblasts observed after burn injury in rats from the EGF + TRF group.

The wound healing process in a patient with diabetes mellitus is impaired due to chronic inflammation, loss of function of macrophages, reduced angiogenesis, stiffer blood vessels and poor maturation and strengthening of tissues^55^ and over‐expression of matrix metalloproteases (MMPs).^56^

**FIGURE 4:**
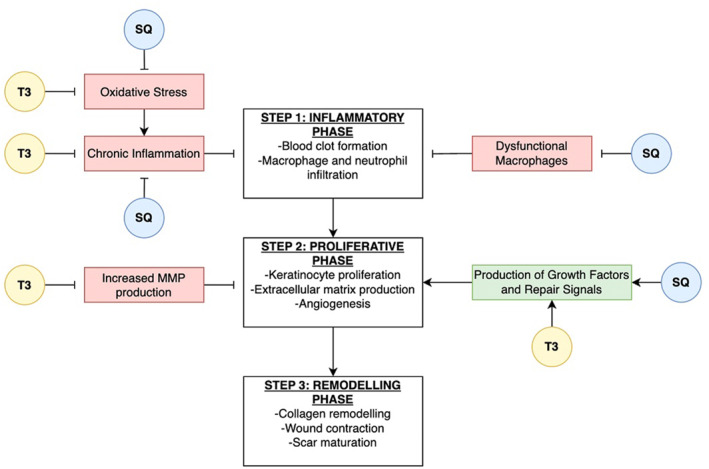
Simplified diagram that shows key steps of wound healing (white) and their stimulating (green) and inhibitory (red) factors modulated by tocotrienols (yellow) and squalene (blue). MMP, matrix metalloproteinases; T3, tocotrienol; SQ, squalene; →, stimulation; ⊥, inhibition.

Topical application of TRF to full‐thickness skin excision wounds in type 2 diabetic (T2D) mice resulted in a notably faster rate of wound closure and healing when compared to an untreated control group. When TRF was topically applied to subjects with T2D, an elevation in IL‐4 levels and suppression of pro‐inflammatory cytokines and chemokines, such as IL‐1α, IL‐17A, leukaemia inhibitory factor, growth regulated oncogene, macrophage inflammatory proteins (MCP‐1, MCP‐3), regulated upon activation, normal T cell expressed and presumably secreted interferon gamma‐induced protein, and granulocyte‐macrophage colony‐stimulating factor (GM‐CSF) was observed^57^ (Figure 4). Furthermore, the study indicated a reduction in oxidative stress, as reflected by decreased myeloperoxidase levels and increased glutathione peroxidase and catalase activity. Interestingly, the research also assessed the production of MMP‐9 in diabetic wounds, and TRF‐treated animals showed a significant reduction in MMP‐9 production, which enhanced the diabetic wound‐healing process.^57^

In a separate investigation, Chong and colleagues assessed the efficacy of T3‐rich nanoemulsions (NE) derived from red palm oil on wound healing. The initial segment of the study involved in vitro assessment using keratinocyte cell lines, employing a scratch migration assay. A recent study showed that a T3‐rich nano‐emulsion formulation exhibited a higher gap closure rate in a scratch migration assay using keratinocyte cell lines, which a commonly used cell‐based model for wound healing compared to untreated cells.^58^ In the same study, the authors also showed that zebrafish with tail amputations that were fed with the T3‐rich nano‐emulsion exhibited a remarkable 40%–60% increase in tail regeneration rate compared to the untreated and ascorbic acid‐treated groups.

### 4.4 Tocotrienol protects skin barrier

Skin barrier serves as the first line defence against external threats such as infectious agents, allergens, and toxins. The skin maintains the moisture and transepidermal water level naturally via homoeostasis to prevent excessive water loss. Hence, any impairment of the skin barrier can lead to heightened transepidermal water loss and stimulation of nerve fibres associated with itching. The cycle of itching and scratching can continuously exacerbate damage to the skin barrier, culminating in inflammation.^59^ In a recent study, TRF was reported to protect human epidermal keratinocytes against urban particulate matter, where exposure to TRF reinstated cell viability and mitigated the elevated levels of ROS, and suppressed the activation of the MAPK pathway in keratinocytes stimulated by particulate matters.^33^ The authors also reported reduction in COX2 levels, a downstream inflammatory mediator, and increased the levels of filaggrin, transglutaminase‐1 and involucrin, which shows that TRF safeguarded the skin's barrier functions.

The dermis, the skin's second layer, primarily comprises an extracellular matrix (ECM) called collagen, which is formed by a specialised type of cells referred to as fibroblasts. Collagen (COL) serves as a crucial protein for maintaining the integrity of the skin barrier. A reduction in collagen levels may result in skin losing its epidermis strength as well as elasticity and thickness.^60^ Treating skin fibroblasts with TRF was reported to enhance the expression of genes responsible for coding the COL I and COL III proteins. The COL I and COL III proteins are vital structural components abundant in connective tissues, playing key roles in preserving skin elasticity and integrity. However, as individuals age, collagen production by fibroblasts naturally declines, leading to the formation of wrinkles and a loss of skin elasticity over time.^61^ Therefore, by bolstering collagen synthesis, TRF may help mitigate the effects of ageing on the skin, promoting a more youthful appearance and maintaining skin resilience. Hence, application of TRF in skin cosmetic holds promise in preserving skin health by stimulating collagen production, and also in preventing age‐related phenotypic changes.

## 5 THE ORIGIN AND CHEMISTRY OF SQUALENE

**FIGURE 5:**
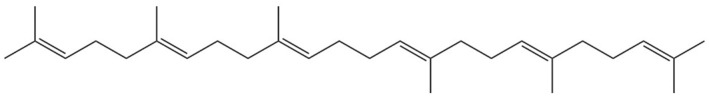
Chemical structure of squalene.

Squalene (SQ) is a naturally occurring triterpene that was first discovered in the liver of sharks (*Squalus Sp*). The chemical structure of SQ consists of 2,6,10,15,19,23‐hexamethyltetracosane with double bonds at positions 2, 6, 10 14, 18 and 22 (Figure 5). In nature, SQ is found in palm oil, amaranth oil and wheat‐germ oil.^62^ SQ constitutes about 13% of the lipids on the skin's surface, commonly known as sebum, and is transported in the bloodstream by very low‐density lipoproteins and distributed in various human body tissues.^63^ Under normal conditions, SQ is concentrated in the sebaceous glands, which release sebum into hair follicles. In addition to SQ, human sebum contains wax esters (25%), cholesterol (2%), triglycerides, and free fatty acids (57%).^64^ The primary function of sebum is to provide lubrication to the skin and hair, and are present throughout the skin, except on the palms of the hands and the soles of the feet. Previous studies have shown that SQ promotes skin health through antioxidant, skin hydrating, emollients, anti‐inflammatory and its wound healing properties,^65^ making it a valuable ingredient in many skincare products.^18^

### 5.1 Effects of squalene on skin erythema

Skin erythema is a medical term used to describe skin redness. The degree of erythema can vary from mild pink or red discolouration to more severe and pronounced redness, depending on the underlying cause and the individual's skin sensitivity.^66^ UVR can potentially induce erythema in sensitive individuals. As a matter of fact, erythema is a marker for assessing sunscreen's sun protection factor (SPF).^67^ A recent study reported that SQ on the skin surface can undergo photooxidation, forming SQ‐monohydroperoxide isomers.^68^ The build‐up of SQ‐monohydroperoxides on skin has been linked to the development of comedones. However, it is worth noting that the comedogenicity of SQ itself is lower when compared to SQ‐hydroperoxide and SQ‐monohydroperoxide.^69^

Topical application of SQ on erythema‐induced male Wistar rats caused marked reductions in the erythema score, and the reduced skin erythema by SQ was correlated with a substantial reduction in the production of O_2_^¯^ in cultured keratinocytes.^70^ In a clinical study, SQ intake in human subjects was observed to significantly reduce facial wrinkles, erythema and pigmentation, and skin samples from the SQ‐supplemented subjects contained high procollagen type I mRNA levels.^71^ Procollagen type I produced by fibroblasts specialised cells is involved in the synthesis of various components of the ECM.^72^ It is a precursor molecule to collagen type I, a major component of the ECM in connective tissues such as skin, tendons, bones and ligaments. Hence, increased expression of collagen by squalene may contribute resilience and resistance to stretching, promote a supportive framework and facilitate wound closure and skin regeneration.

### 5.2 Squalene in wound healing

One of the crucial characteristics of inflammatory skin reactions is the macrophages, which play a central role in innate and adaptive immune responses. Macrophages are activated by infection, injury, trauma, heat and radiation. Activated macrophages can differentiate into M1 and M2 phenotypes, depending on stimuli. Activated M1 macrophages are pro‐inflammatory and secrete cytokines (e.g. IL‐1β, IL‐6, IL‐18, TNFα) that are associated with defence mechanism. The M2 macrophages transition from the M1 phenotype, provide anti‐inflammatory effects and modulate tissue remodelling and wound healing.^73^, ^74^ Recent evidence showed that SQ can modulate macrophage response to inflammation and drives towards the wound healing process. For instance, exposing human leukaemia monocytic cell line (THP1 cells) with SQ demonstrated higher anti‐inflammatory properties at lower concentrations of SQ as THP1 cells treated with different concentrations of SQ exhibited an increase in IL‐10, IL‐13 and IL‐4, which are the anti‐inflammatory cytokines and suppressed pro‐inflammatory mediators (TNF‐α and NF‐κB).^75^ In addition, SQ also enhanced the signals to induce migration of eosinophils and neutrophils migration to the wound site, as well as promoted remodelling and repairing signals such as activating TIMP metallopeptidase inhibitor 2, eotaxin‐2, granulocyte colony‐stimulating factor and GM‐CSF.^75^

Recently, it was reported that treatment with a topical emulgel using an agar base loaded with SQ through a physical cross‐linking technique demonstrated favourable granulating tissue, efficient epithelialisation and reduced wound diameter, leading to uniform scarless tissue growth.^76^ The histopathological analysis revealed that the formulation expedited the neovascularisation process, accompanied by significant collagen formation, decreased CD68 macrophages and accelerated keratinocyte proliferation. In a separate study, a formulation that coupled SQ with vitamin C showed increased epidermal thickness and collagen III production via increased glycosaminoglycans on human skin explants.^77^ These studies show good evidence that SQ has significant potential to treat skin conditions associated with inflammation and facilitate wound healing process through multiple signalling pathways (Figure 4).

## 6 CONCLUSIONS

In conclusion, the exploration of tocotrienols and squalene in this review article highlights their multifaceted and promising roles in addressing various skin conditions. Tocotrienols emerged as versatile allies in the quest for optimal skin health, and their protective prowess extends beyond the superficial, demonstrating notable benefits in shielding against dermatological challenges and promoting recovery from wounds. Similarly, the squalene shed light on its significance, particularly in addressing skin erythema and playing a constructive role in wound healing. The effects of squalene on the skin's inflammatory response and its contribution to the intricate process of wound repair were detailed, emphasising its relevance in dermatological contexts. Collectively, the findings presented in this review underscore the promising potential of both tocotrienols and squalene as therapeutic agents for a spectrum of skin conditions. Their multifaceted protective mechanisms, ranging from mitigating inflammation to enhancing wound healing, position them as compelling candidates for further exploration in dermatological research and applications. As we navigate the evolving landscape of skincare, the continued investigation and utilization of tocotrienols and squalene hold the promise of innovative and effective interventions for diverse skin concerns.

## CONFLICT OF INTEREST STATEMENT

The authors declare that they have no conflict of interest.

## AUTHOR CONTRIBUTIONS

**Nevvin Raaj Morgan**: Formal analysis (equal); investigation (equal); visualization (equal); writing – original draft (equal). **Kasthuri Bai Magalingam**: Conceptualization (equal); data curation (equal); project administration (equal); supervision (equal); writing – review & editing (equal). **Ammu Kutty Radhakrishnan**: Conceptualization (equal); funding acquisition (equal); project administration (equal); supervision (equal). **Mohan Arumugam**: Supervision (equal). **Adawiyah Jamil**: Supervision (equal). **Saatheeyavaane Bhuvanendranpillai**: Supervision (equal).

## ETHICS STATEMENT

This article does not contain any studies with human participants or animals performed by any of the authors.

## CONSENT TO PARTICIPATE

Corresponding and all the co‐authors are willing to participate in this manuscript.

## CONSENT FOR PUBLICATION

Corresponding and all the co‐authors are willing to participate in this manuscript.

## Data Availability

Despite the provision of sufficient data through figures, the authors affirm that should additional data be necessary, it will be made available upon request.
